# The effect of online video-assisted teaching program on medical students learning procedure of fractional curettage

**DOI:** 10.1186/s12909-023-04052-3

**Published:** 2023-02-02

**Authors:** Oracha Chucherd, Sakda Arj-Ong Vallibhakara, Krissada Paiwattananupant, Pongtong Puranitee, Rujira Wattanayingcharoenchai, Orawin Vallibhakara

**Affiliations:** 1 Department of Obstetrics and Gynaecology, Faculty of Medicine, Ramathibodi Hospital, Mahidol University, Bangkok, 10400 Thailand; 2 Faculty of Medicine, BangkokThonburi University, Bangkok, 10170 Thailand; 3 Child Safety Promotion and Injury Prevention Research Center, Faculty of Medicine, Ramathibodi Hospital, Mahidol University, Bangkok, 10400 Thailand; 4 Department of Obstetrics and Gynaecology, Gynecologic Oncology Unit, Faculty of Medicine, Ramathibodi Hospital, Mahidol University, Bangkok, 10400 Thailand; 5 Department of Pediatrics, Faculty of Medicine, Ramathibodi Hospital, Mahidol University, Bangkok, 10400 Thailand; 6 Department of Obstetrics and Gynaecology, Female Pelvic Medicine and Reconstructive Surgery Unit, Faculty of Medicine, Ramathibodi Hospital, Mahidol University, Bangkok, 10400 Thailand; 7 Department of Obstetrics and Gynaecology, Reproductive Endocrinology and Infertility Unit, Faculty of Medicine, Ramathibodi Hospital, Mahidol University, Bangkok, 10400 Thailand

**Keywords:** Medical education, Online video-assisted teaching, Fractional curettage, Online learning, Undergraduate, Lecture videos, Teaching method

## Abstract

**Objectives:**

Since 2020, with the entire world in crisis over the coronavirus pandemic (COVID-19), medical students have adapted to hybrid and distance learning. This study aims to compare the learning outcomes of students learning the procedure of fractional curettage in an online video-assisted teaching program to those of students learning the procedure in a traditional class.

**Methods:**

A quasi-experimental study was conducted among fourth-year medical students who rotated to Obstetrics and Gynecology courses between April 2021 and October 2021. Participants in the first two rotations were enrolled in traditional classes, and the online video-assisted teaching program was introduced in the subsequent two rotations. Both study groups took OSCEs (objective structured clinical examinations), a pre-test and post-test with MCQs (multiple choice questions), and a confidence and satisfaction level questionnaire.

**Results:**

A total of 106 fourth-year medical students, 54 in the traditional group and 52 in the online video-assisted teaching program, were recruited. The online video-assisted group showed a statistically better mean OSCE score (85.67 ± 11.29 vs. 73.87 ± 13.01, *p* < 0.001) and mean post-test MCQ score than the traditional group (4.21 ± 0.87 vs. 3.80 ± 0.98, *p* = 0.0232). Moreover, the mean difference between the two groups' pre and post-test MCQ scores was significantly different (0.96 ± 1.37 vs. 1.79 ± 1.50 in traditional and online video-assisted teaching program groups, respectively, *P* = 0.0038). The participants in the experimental group reported significantly greater confidence (*P* < 0.001) in performing the fractional curettage procedure. However, the mean satisfaction score was significantly higher in the control group (*p* = 0.0053).

**Conclusion:**

The online video-assisted teaching program on the fractional curettage procedure, a necessary and skill-demanding procedure, is an effective and advantageous education tool that improves skills, knowledge, and confidence in fourth-year medical students. We recommend that the video-assisted teaching program is another effectively procedural teaching method for medical students.

## Introduction

Fractional curettage is a fundamental procedure in Obstetrics and Gynaecology by which a sample of the endocervix and endometrium tissue is taken from the uterine cavity [1]. Medical schools have demanded that this procedure be taught since the introduction of Thailand's licensing regulations for physicians. In the Department of Obstetrics and Gynaecology, medical students gain the knowledge and skills required to perform the intervention for patients safely. They must be able to perform the procedure, describe steps, list contraindications, and treat complications. Therefore, medical schools must offer students learning resources to support their self-study of clinical skills [2]. In Thailand, at the Faculty of Medicine, Ramathibodi Hospital, Mahidol University, Bangkok, medical students receive the first systematic instruction and teaching on fractional curettage in the fourth year. (the first year of their clinical clerkship). The instruction by Obstetrics & Gynaecology attending physicians involves demonstrations done in a small group class (approximately 10–14 students) by using a model, IUD training model (half) OG032 (Brilliant Rubber Co., Ltd. Songkhla, Thailand). Since 2020, Thailand has faced the coronavirus pandemic (COVID-19) crisis. The increasing number of cases prompted the government to declare a state of emergency on March 26, 2020, enforcing strict city lockdowns, which closed all restaurants, stores, and entertainment venues, with exceptions for essential services [3]. Local and international schools, universities, and children's care facilities were closed to impede the spread of COVID-19 [4]. Medical schools had to adapt from a small on-site group class to distance learning over a video conferencing system. Patient numbers decreased during the pandemic, and the lockdown policy limited on-site time. The COVID-19 pandemic measure affected lesser clinical experience and clinical exposure among medical students. To close this gap, the medical education team turned to online video-assisted teaching, assuming it would benefit procedural learning such as fractional curettage, an essential fundamental procedure that medical students must know. Moreover, the other advantages of online-teaching materials are that they provide a clear visualization of the procedure in a realistic setting, step-by-step, and are convenient to access on demand everywhere and every time. A particular benefit during this COVID Pandemic crisis is promoting the new normal learning through digital transformation to provide several advantages. The lectures can be viewed at any time, paused when finding more information, repeated as needed, and adjusted to playback at the individual's preferred speed [5].

In comparison, traditional lectures are limited by the lecturers' skills, inability to repeat the classes, and limited educational material usage. However, small in-person classes provide a familiar teaching and learning, allowing for much more two-way communication and enabling students to ask questions promptly during online classes [6]. Then, we hypothesized that the online video-assisted teaching program would enable medical students to perform fractional curettage as accurately as the traditional class. The primary objective was to compare students' performance of the fractional curettage procedure, as evaluated by objective structured clinical examination (OSCE) as the gold standard, after taking either a traditional or an online video-assisted class. The secondary objective aimed to compare knowledge after the course (measured by multiple-choice questions (MCQs) post-test score), the confidence to perform the procedure, and satisfaction with students' learning in the online video-assisted teaching program to students who are in the traditional class.

## Materials and methods

### Populations

A quasi-experimental study was conducted at the Department of Obstetrics & Gynaecology, Faculty of Medicine Ramathibodi Hospital, Mahidol University, between April 2021 and October 2021 in accordance with the TREND statement [7]. The study was conducted on first-semester, fourth-year medical students who rotated to the course of Obstetrics and Gynaecology during the academic year of 2021. This research protocol was approved by Human Research Ethics Committee, Faculty of Medicine Ramathibodi Hospital, Mahidol University (COA. MURA2021/295). Institutional Review Boards in Mahidol University are in full compliance with International Guidelines for Human Research Protection such as Declaration of Helsinki, The Belmont Report, CIOMS Guidelines and the International Conference on Harmonization in Good Clinical Practice (ICH-GCP) The study protocol was also submitted to the Thai Clinical Trials Registry; TCTR (www.thaiclinicaltrials.org) Clinical trial registration no. TCTR20221014006/14/10/2022.There was no conflict of interest. Informed consent was obtained from all participants before entering this study. All methods were carried out in accordance with Declaration of Helsinki. The group was selected randomly for convenience from the Faculty of Medicine, Ramathibodi Hospital, Mahidol University. Since this study aimed to examine the effectiveness of an educational intervention in an actual setting; a random assignment or prevention of intervention contamination was not possible to apply. Thus, a non-equivalent group design was applied according to the convenient time sequence of clerkship rotation. The inclusion criteria were medical students taking part in the Obstetrics & Gynaecology rotation, willing to participate, and having never been exposed to any class or demonstration on fractional curettage procedure before. Students who could not follow the study protocol or be unwilling to participate in this study were excluded. Participants were informed that they could leave this study at any time and the evaluation component would not influence their final grades.

## Methods

Fourth-year students were allocated to the traditional group (the first and the second group of the academic year) and the online video-assisted teaching program (the third and the fourth group of the academic year). The students in both groups rotated in the Obstetrics and Gynaecology department for 4 weeks. All the participants were informed about the research project in the introductory session of their fourth-year Obstetrics and Gynaecology rotation. After the enrollment, informed consent was obtained, and a 5 MCQ pre-test covering the knowledge of indication, contraindications, steps, and complications of fractional curettage procedure was evaluated. Participants' demographic information and characteristics regarding age, sex, and grade point average (GPA) were collected.

### Interventions

#### Traditional class

Participants allocated to the traditional class were divided into a small group of 10–14 students per class and taught about the indication, contraindications, steps, and complications of fractional curettage procedure by Obstetrics & Gynaecology attending physicians with the hands-on model for 60 min in the second week of the student rotations. Initially, attending physicians demonstrated the steps of the fractional curettage procedure in front of the classroom. After that, each student came out to perform the procedure under supervision, and received feedback from an attending physician during the process. Moreover, after the class, students can bring models for more self-practicing.

### Online video–assisted teaching program

All participants allocated to the experimental group accessed an online video of the procedure of fractional curettage that was uploaded to the electronic learning website (E-learning program) of the Department of Obstetrics & Gynaecology, Faculty of Medicine, Ramathibodi hospital on the first day of their rotations. The video described the indication, contraindications, steps, and complications of the fractional curettage procedure [8]. The footage contained sections describing the steps of fractional curettage, including preparation, speculum insertion, endocervical curettage, cervical dilatation, uterine sound, and endometrium curettage. The procedure was performed by a professional Obstetrics & Gynaecology physician on an actual patient presenting with postmenopausal bleeding. The patient consented to participate in the demonstration without being identified in the video. The video was created under the supervision of an Obstetrics & Gynaecology professional having expertise in voice-over and narration in Thai. The 5 Fr. diagnostic hysteroscope (KARL STORZ, Germany) was used to show the visible cervix, a difficult feat to achieve using traditional demonstrations with pelvic models. Students not only watched the fractional curettage procedure in a real patient and saw the endometrial tissue obtained, but they were also able to observe the manner in the operating room. Another assignment was recording a video of themselves performing the fractional curettage procedure on the same model in the traditional class group and sending it to attending physicians. This usually took more time to practice compared to traditional classes. Written feedback from medical staff was returned to everyone via email by the second week of their rotations.

### Outcome measurement

In both groups, the main outcomes were measured in the third week of rotations. Student performance in the procedure of fractional curettage was assessed by a five-minute individually validated objective structured clinical examination (OSCE). The OSCEs were evaluated blindly by two Obstetrics & Gynaecology attending physicians, and points were awarded from 0–100 (30 for preparation, 60 for skill and techniques, and 10 for post-procedure examination). Next, a 5-MCQ post-test was given, and students were asked to report their level of satisfaction with learning and confidence in performing fractional curettage. The primary outcome of the study was the OSCE score. The secondary outcomes were (1) the score on the MCQ post-test (0–5 points possible), (2) the level of confidence in performing fractional curettage on a 5-Likert scale from 1 to 5 (1 = least confident, 5 = most confident), and (3) the level of satisfaction in learning fractional curettage on a scale of 1 to 5 (1 = least satisfaction, 5 = most satisfaction).

MCQ and OSCE criteria were prepared in accordance with the lesson plans for medical students of Obstetrics & Gynaecology, Faculty of Medicine Ramathibodi Hospital. Ten questions were written for the MCQ tests and separated randomly into pre-test and post-test groups. The questions were based on main content items, including indication, contraindication, techniques, steps, post-procedure examination, and complications of the fractional curettage procedure.

**Fig. 1:**
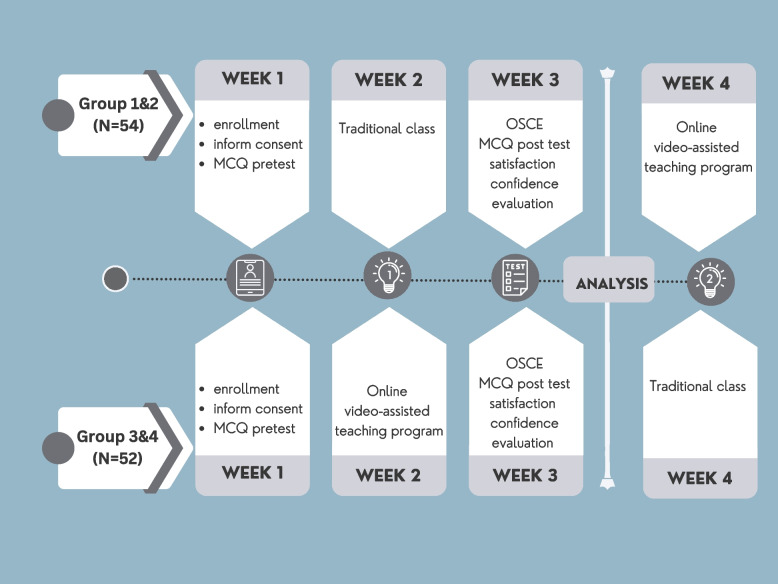
Study methods in the traditional group and the online video-assisted teaching group. Abbreviation: MCQ; multiple choice question, OSCE; objective structured clinical examinations

In the fourth week of their rotations, the traditional group received the online video-assisted teaching program on fractional curettage and vice versa. The online video-assisted group also received the traditional class experience by attending physicians' demonstrations of fractional curettage procedures with a model. That both the control and the experimental group received the same learning experience before the end of the rotation as shown in Fig. 1.

### Data analysis and statistical methods

The sample size was calculated by two independent means formula. The power of the study was designed to be greater than 90%, with a P value below 0.05 considered statistically significant. Based on the data from a previous study by Sarihan A, et al*.* that compared traditional lectures to video-supported lectures in emergency medicine training in Turkey [6]. Therefore, the calculated number of participants needed was 51 in each group. Baseline characteristics were reported as descriptive data. All quantitative variables were evaluated for normal distribution, mean ± standard deviation (SD) and median (range) were used when data showed normal and non-normal distribution, respectively. The statistical analyses were performed by Stata Statistical Software, version 16.0 (StataCorp LLC, College Station, TX, USA). The statistical significance of the present study was defined as a P value less than 0.05 with a 95% confidence interval (CI).

## Results

A total of 106 fourth-year medical students in the four Obstetrics & Gynaecology clerkship rotations were enrolled. The total number of participants in the controlled (traditional class) and experimental group (online video-assisted teaching program) was 54 and 52, respectively. The median age of the participants was 21 years old, with 51.8% male. There was no statistically significant difference in baseline characteristics between the two groups, including median age, gender, and median grade point average (GPA). The median GPAs were 3.52 (2.45, 3.97) and 3.61 (2.18, 3.95) in the traditional group and online video-assisted teaching program group, respectively. In comparison, the mean MCQ pre-test scores were higher in the traditional group compared to the online video-assisted teaching program group, 2.83 ± 0.95 and 2.42 ± 0.87, *p* = 0.06, respectively, as shown in Table 1.

**Table 1:** Baseline characteristics of the participants

Characteristics	Traditional Class (*N* = 54)	Online Video-assisted Teaching program (*N* = 52)	*p*-value
Age	21(20,25)	21(20,29)	0.067
GPA	3.52 (2.45,3.97)	3.61 (2.18,3.95)	0.174
Gender^a^			0.692
Female	27 (50)	24 (46.15)	
Male	27 (50)	28 (53.85)	
Pre-test MCQ score^b^	2.83 ± 0.95	2.42 ± 1.26	0.06

Data were shown as median (range), ^a^ n (%), and ^b^ mean ± SD

*Abbreviation*: *GPA* Grade point average, *MCQ* Multiple choice question

The online video-assisted teaching program produced statistically significantly better outcomes in almost all aspects of medical students' learning of fractional curettage including OSCE score, MCQ post-test score, and confidence to perform the procedure. The exception was learning satisfaction, as shown in Table 2. The mean OSCE scores were significantly higher in the online video-assisted teaching program group compared to the traditional group, 85.67 ± 11.29 and 73.87 ± 13.01, *p* < 0.001, respectively. In addition, the mean post-test MCQ score of the online video-assisted teaching program group was statistically higher than the traditional group, 4.21 ± 0.87 and 3.80 ± 0.98, *p* < 0.05, respectively. The results of the participant questionnaire after the Obstetrics and Gynaecology rotation showed participants in the online video-assisted teaching program group reporting statistically significant greater confidence in performing fractional curettage procedures, 3.63 ± 0.627 and 3.30 ± 0.69, *p* < 0.01, respectively. However, the mean satisfaction score in learning experience was significantly higher in the traditional group compared to the online video-assisted teaching program group, 4.22 ± 0.74 and 3.79 ± 0.82, *p* < 0.01, respectively. Moreover, the mean difference between the two groups pre-test and post-test MCQ scores were significantly different, 0.96 ± 1.37 and 1.79 ± 1.50, in the traditional and online video-assisted teaching program groups, respectively (*p* < 0.01), as shown in Table 2.

**Table 2:** Outcomes after intervention between the traditional group and the online video-assisted teaching group

Outcomes	Traditional Class	Online Video-assisted Teaching program	*p*-value
OSCEs score (0–100)	73.87 ± 13.01	85.67 ± 11.29	< 0.001*
Post-test MCQ score (0–5)	3.80 ± 0.98	4.21 ± 0.87	0.0232*
Learning Satisfaction (0–5)	4.22 ± 0.74	3.79 ± 0.82	0.0053*
Confidence to perform the procedure (0–5)	3.296 ± 0.69	3.63 ± 0.63	0.0096*
Mean difference of pre-test and post-test MCQ score	0.96 ± 1.37	1.79 ± 1.50	0.0038*

Data were shown as mean ± SD

^*^ Statistically significant

## Discussion

Fractional curettage is a basic procedure necessary for general physicians to practice and is one of the level II procedures according to Thai Medical Competency Assessment Criteria for National License 2012 [9]. Before the COVID-19 pandemic, at the Faculty of Medicine Ramathibodi Hospital, medical students were taught how to perform this procedure by Obstetrics & Gynaecology staff demonstrating in 60-min small classes of 10–14 students with hands-on practice on a pelvic organ model under direct observation. After the social distancing situation, we conducted a quasi-experimental study to evaluate the effects of introducing an online video-assisted program to teach fractional curettage to fourth-year medical students. Our online video-assisted teaching program contained content designed to fulfill the lesson plan's objectives with a clear and realistic demonstration of a consenting patient. This study demonstrated that the medical students who experienced the online video-assisted teaching program had significantly better OSCE scores on the fractional curettage procedure than traditional classes. One of the reasons is the practicing and self-study time before the performance, which is supposed to be much more in the online video-assisted teaching program group than in the traditional class. More studying and practicing time encourage the "knows-how" and "show how" in Miller's assessment pyramid [10]. This was in line with a previous study on 30 emergency residents learning about Advantage Trauma Life Support by Sarihan A, et al. [6], which reported that students receiving video-supported lectures achieved significantly higher OSCE scores compared with those receiving only traditional lectures. Farahmand S, et al. [11] evaluated the effect of distance learning by 50-min Digital Video Disc (DVD) vs. traditional 50-min lecture on the performance of the initial assessment and management in trauma patients by 120 senior medical students who also did a hands-on session on a mannequin for another 50 min. They reported that the distance learning group performed statistically significantly better (*P* < 0.001) on their OSCEs. In contrast, Schwerdtfeger K, et al. [12] found no statistically significant difference in OSCE scores between the traditional class group and the video-assisted group in a sample of 256 medical students learning the principles of the acute treatment of trauma patients in a multidisciplinary course on emergency and intensive care medicine. However, the video-assisted group showed better global performance and more relative knowledge increase than the traditional class.

Our results found that, in addition to better OSCE scores, the online video-assisted teaching program group achieved higher mean post-test MCQ scores and higher confidence in performing fractional curettage procedures than the traditional group. According to Mayer’s cognitive learning theory, video-assisted teaching programs support the learning environment, both visually and auditory, more than traditional classes [13]. Video-assisted teaching is convenient, easy to access, and delivers consistent quality independent of the person’s teaching skills. It also allows students to repeat the learning experience at their own pace and makes the details of the fractional curettage procedure more understandable through a multitude of effects, including sound effects, superimposed text, graphic illustrations, and picture-in-picture insertions along with narrative sound [14]. The video-assisted teaching program provided optimal observation points so that all medical student viewers got the same experience and standard.

On the contrary, traditional classes provided different vantage points in each class depending on the students' and teachers' positions, classroom layout, and space and time constraints. It was difficult to observe the demonstration for some students, especially due to the small and narrow space in the pelvic organ. Moreover, listening comprehension may suffer in the traditional class when listeners lose their attention and have no chance to revise. Despite these drawbacks, students learning fractional curettage in the traditional classroom were more satisfied, possibly because they could easily ask impromptu questions, had deeper, more enriching discussions, and had better interpersonal interactions with their attending physicians and peers. Moreover, satisfaction might depend on the year of study of the population. Since the medical students in our population were studying in their fourth year, which is the first year of clinical experience, they were likely to place a higher value on personal engagement with their attending physicians.

Video-assisted teaching programs enable learners to gain practical and standardized experience and develop required clinical skills. The results may depend on the quality and content of the video. In our study, the video employed camera angles that made the anatomy of the vagina and cervix visible, which is difficult to do in real experience, and used clear narrative sound created under professional supervision to describe the steps of the procedure. Our study provides convincing evidence that the video-assisted teaching program was well received by students and is a convenient teaching medium, not inferior to the traditional class. Relevantly, a study by Carina Bachmann, et al. shows that using online material to prepare students' knowledge and pique their curiosity before beginning face-to-face classes promotes the type of "blended learning" setting that fosters student and teacher engagement in the question-asking behaviors, discussion, and practicing with minimal variability in teaching content. This has important implications for the instructional design of both the online and in-person learning experience [15, 16]. Our results confirm that video-assisted teaching programs benefit clinical and procedural skills, especially in the gynecologic and obstetrics field, which deals with a private area where the anatomy is difficult to distinguish by sight. Therefore, we encourage using online videos as effective medical education material, especially for standard gynecologic and obstetrics procedures.

### Strengths & limitations

Although many previous studies focused on the effects of video-supported learning of medical procedures, this study was the first that focused on the fractional curettage procedure using an online video-assisted teaching program and measuring the performance and knowledge outcomes of medical students. This medical school study followed a quasi-randomized design with assignments based on enrollment in a rotation, a design which allowed all of the students in each rotation to freely share their learning materials e.g., the video on fractional curettage without causing contamination. The study population in both groups had no statistically significant difference in baseline characteristics. Moreover, our study assessed outcome in multiple dimensions: objectively, by measuring procedural skill as OSCE score and knowledge as MCQ score, and subjectively, through its evaluation of satisfaction with the learning experience and confidence to perform the procedure after the end of the rotation. The students could not be blinded to the intervention because this study was implemented in a real education setting, and the evaluator was not blinded. However, observer bias was minimized by using a validated instrument and controlled rater training. The OSCE scores were independently evaluated by two Obstetrics & Gynaecology attending physicians, while the MCQ score, level of confidence, and level of satisfaction were non-operator dependent assessments.

### Application

Online video-assisted teaching is an effective tool for teaching medical procedural skills. In addition, online video-assisted teaching may be used in conjunction with traditional class learning or adjunct tools before the hands-on experience to create blended learning, which may be more effective than traditional class alone.

### Future studies

Firstly, the knowledge and skill retention should be evaluated, the procedure practicing results in the muscle-memory, which might effect on the greater knowledge and skill retention. We suggest that prospective randomized controlled studies be undertaken to prove the effectiveness of online video-assisted teaching programs on other procedural skills training, such as marsupialization, vaginal delivery, and vacuum extraction. Moreover, further studies could be conducted at different levels of medical training, such as resident and fellowship training. We also recommend a study comparing "blended teaching programs," which combine traditional classes with video-assisted teaching to traditional-only programs. This research will provide valuable insight into the design of effective medical education programs that improve both students' knowledge and clinical skills.

## Conclusion

The online video-assisted teaching program on fractional curettage procedure is an effective tool that enabled fourth-year medical students to achieve higher OSCE scores, MCQ scores, and confidence to perform the procedure than those who received traditional instruction.

## Data Availability

All data and materials can be made available to the journal on request. Request for data can be submitted to the corresponding author by email orawin.val@mahidol.ac.th.
